# Comparing feasibility of low-tube-voltage protocol with low-iodine-concentration contrast and high-tube-voltage protocol with high-iodine-concentration contrast in coronary computed tomography angiography

**DOI:** 10.1371/journal.pone.0236108

**Published:** 2020-07-16

**Authors:** Min Jae Cha, Sung Mok Kim, Tae Ran Ahn, Yeon Hyeon Choe

**Affiliations:** 1 Department of Radiology, Samsung Medical Center, Sungkyunkwan University School of Medicine, Seoul, Republic of Korea; 2 Imaging Center, Samsung Medical Center, Heart Vascular Stroke Institute, Sungkyunkwan University School of Medicine, Seoul, Republic of Korea; University of Oklahoma, UNITED STATES

## Abstract

**Background:**

To investigate the feasibility of a low tube voltage (80 kVp) protocol with low concentration contrast media (CM) (iodixanol 320 mgl/ml) as compared with a high tube voltage (100 kVp) protocol with high concentration CM (iomeprol 400 mgl/ml) in coronary CT angiography (CCTA) for patients with body mass index less than 30.

**Materials and methods:**

A total of 93 patients were randomly assigned into three groups and underwent CCTA as follows: Group A) 100 kVp, 100–350 mAs, 400 mgl/ml CM at 4ml/s, and reconstructed with filtered back projection; Group B and C) 80 kVp, 100–450 mAs, 320 mgl/ml CM at 4 ml/s and 5 ml/s, respectively and reconstructed with iterative reconstruction. Objective and subjective image quality (IQ) was analyzed.

**Results:**

The image noise, intravascular attenuation, signal-to-noise ratio and contrast-to-noise ratio of major coronary arteries did not differ significantly among three groups. Subjective IQ analyses on vascular attenuation and image noise did not differ significantly, either (all of *p* > 0.05). Qualitative IQ of Group B and C was non-inferior to that of Group A. Substantial reduction of radiation exposure was achieved in group B (2.60 ± 0.48 mSv) and C (2.72 ± 0.54 mSv), compared with group A (3.58 ± 0.67 mSv) (*p* < 0.05).

**Conclusion:**

CCTA at 80 kVp with 320 mgl/ml CM and iterative reconstruction is feasible, achieving radiation dose reduction, while preserving IQ.

## Introduction

With advances of acquisition techniques of computed tomography (CT), coronary computed tomography angiography (CCTA) has been widely used as a reliable imaging modality for assessing coronary artery disease (CAD). However, CCTA has inevitable drawbacks, including the exposure to radiation and iodinated contrast media (CM), which carries potential risks for cancer and contrast-induced nephropathy. As with other CT applications, it is apparent that higher dose of radiation and higher concentration of iodinated CM can provide images with lesser noise and higher contrast, respectively [1, 2]. Consequently, various studies were performed to reduce the radiation exposure and amount of iodinated CM while preserving image quality (IQ), referring to the so-called ‘as low as reasonably achievable’. Particularly, numerous studies have reported advantages of low x-ray tube energy, which can reduce both radiation exposure and amount of contrast agent [3–5]. In fact, the decreased contrast effect due to reduction of iodinated CM can be compensated with the low tube potential, because it shows high iodine contrast at low photon energies. Additionally, various iterative reconstruction (IR) techniques had been developed, and substantial noise reduction was achieved without IQ degradation for low dose CCTA [6, 7]. However, there are still limited reports on lowering the tube voltage to 80 kVp and iodine concentration of the CM for CCTA.

We hypothesized that the CCTAs performed using a low-tube-voltage (80 kVp) protocol with low-iodine-concentration CM (iodixanol 320 mgl/ml) and IR algorithm could provide non-inferior IQ, as compared to CCTAs with high-tube-voltage (100 kVp) protocol with high-iodine-concentration agent (iomeprol 400 mgl/ml) and conventional filtered back projection (FBP) technique. Hence, we performed a prospective study to investigate the feasibility of performing CCTA at 80 kVp with 320 mgl/ml of CM with IR by performing subjective and objective IQ analyses.

## Material and methods

The institutional review board of Samsung Medical center approved this prospective study (IRB-2013-10-084-015), and patient written informed consent was acquired at the time of the CCTA was ordered.

### Patient selection

**Fig 1:**
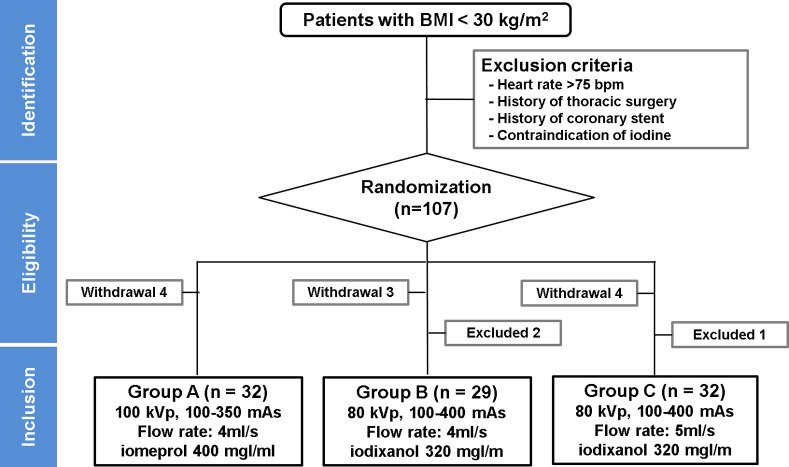
Flowchart illustrating patient enrollment.

Among patients undergoing CCTA to assess CAD, participants who were ≥ 20 years of age with body mass index (BMI) < 30 kg/m^2^ were identified. The exclusion criteria included heart rate over 75 beats/minute, previous history of percutaneous coronary angioplasty or stenting, previous history of thoracic surgery, and contraindications for use of iodinated CM. Overall, 107 eligible participants were randomized into three groups (groups A, B, and C) in a 1:1:1 allocation ratio to one of three arms. Excluding those with heart rate > 75 beats/minute (n = 3) and those who declined to participate in the study during CCTA acquisition (n = 11), 93 subjects (45 men and 48 women; mean age: 50.89 ± 11.51 years) were enrolled in the study with informed consent (Fig 1).

### Contrast media injection protocol

High-iodine-concentration contrast agents (iomeprol 400 mgI/ml; Bracco, Milan, Italia) and low-iodine-concentration contrast agents (iodixanol 320 mgI/ml; GE Healthcare, Giles St Chalfont, United Kingdom) were used in group A and groups B and C, respectively. The CM was administered via the antecubital vein (total amount of contrast injected = 0.9 x body weight [mL]) followed by 30 mL of saline chaser. To minimize the effect of iodine delivery rate, the injection rate of the contrast for group C was elevated to 5 mL/s, as opposed to 4 mL/s for groups A and B. Thus, the iodine delivery rate (iodine concentration multiplied by injection rate) was identical in groups A and C [8].

### CCTA protocol

All CCTA studies were acquired with a 64-slice multidetector CT scanner (Discovery HD 750, GE Healthcare, Milwaukee, WI, USA) using retrospective electrocardiography gated helical mode. Nitroglycerin 0.4 mg was administered sublingually, 1 minute before scanning. The CT image acquisition was initiated automatically once the Hounsfield unit (HU) of the ascending aorta reached 200 HU. To acquire better IQ in each group, scan parameters and reconstruction algorithms were optimized depending on the iodine concentration of the contrast agents. The details of CT parameters and CM were, group A: tube voltage 100 kVp, tube current-time product 100–350 effective mAs, iomeprol 400 mgI/ml at 4 mL/s injection rate; group B: tube voltage 80 kVp, tube current-time product 100–450 effective mAs, iodixanol 320 mgI/ml at 4 mL/s injection rate; and group C: tube voltage 80 kVp; tube current-time product 100–450 effective mAs, iodixanol 320 mgI/ml at 5 mL/s injection rate. All images were reconstructed with FBP and images of groups B and C were additionally reconstructed using an IR algorithm at 50% adaptive statistical IR (ASIR) (GE Healthcare, Waukesha, Wis, USA). The images were then transferred to a picture archiving and communication system (PACS; Centricity 2.0, GE Medical Systems, Mount Prospect, IL, U.S.A.).

### Quantitative image assessment

All images were reviewed and interpreted using PACS workstations. Quantitative analysis of the CT images was performed by two independent and blinded radiologists (with three and 10 years of experience interpreting cardiac images, respectively) with 0.5 mm thick transverse images of the coronary vessel. The coronary artery attenuation values were measured for region of interests (ROIs) in the left main (LM), proximal left anterior descending (LAD), proximal left circumflex (LCx), and proximal right coronary artery (RCA). Additional ROI measurement was performed for an adjacent myocardial fat area.

ROIs were scaled to be as large as possible while excluding the vascular wall, vascular calcification or non-calcified plaque, and artifacts. Intravascular attenuation was measured in HU and vessel noise was defined as the standard deviation of each ROI measurement. The image noise was defined as the standard deviation of the CT attenuation values of the pericardial fat. Three ROI measurements were repeated at each location and averaged to ensure data consistency. The mean attenuation values of the coronary vessels and pericardial fat were calculated by averaging the values obtained from the two observers.

The signal-to-noise ratio (SNR) and contrast-to-noise ratio (CNR) were calculated using the following formulas: SNR = mean coronary attenuation / image noise, CNR = (mean coronary attenuation–perivascular fat attenuation)/ image noise.

### Qualitative image assessment

Subjective IQ of vascular contrast attenuation and image noise were assessed independently by two observers (with three and 10 years of experience interpreting cardiac images, respectively). In discordant scores, a consensus reading was performed between the two observers. The IQ of the attenuation of the coronary was assessed with a 5-point Likert scale as follows: score of 1, insufficient attenuation resulting in a non-diagnostic examination; 2, poor, suboptimal attenuation with low diagnostic confidence; 3, fair, acceptable attenuation of relevant cardiac anatomy and vascular access; 4, good, satisfactory attenuation providing sufficient evaluation of relevant anatomy; 5, excellent, strong attenuation of even the smallest arteries [9]. The presence and extent of image artifact was assessed with the 5-point Likert scale as follows: score of 1, non-diagnostic, severe artifact; 2, poor, substantial artifact but sufficient contrast attenuation for assessing the heart system; 3, fair, moderate artifact not interfering with a comprehensive examination of the coronary artery; 4, good, with only minor artifact not interfering with assessment; 5, excellent, with no artifact or any diagnostic limitation [9].

### Radiation dose

The CT dose index volume and dose length product (DLP) were recorded. Effective dose was obtained by multiplying DLP with the conversion coefficient (k = 0.014 mSv/mGy·cm in the cardiothoracic area).

### Statistical analysis

Sample size calculation was based on a margin of non-inferiority for subjective IQ score of 0.6 [10–12]. The total number of subjects required was 99 (33 per group), including a 10% dropout, in order to obtain a power of 90% and a two-sided α-level of 0.05 to demonstrate the non-inferiority of IQ for the 80 kVp protocol with low-iodine-concentration CM. Continuous variables were expressed as the mean ± standard deviation. Kruskall-Wallis tests were performed to assess differences in demographic data, including age, height, weight, BMI, body surface area, and average heart rate between the three groups. Kruskall-Wallis analyses with Dunn-Bonferroni corrections were applied to compare DLP and effective dose of CCTA, intravascular CT attenuation, image noise, SNR, and CNR between the three groups. Inter-observer agreement was tested by calculating the intraclass correlation coefficient (ICC) for quantitative parameters. Kappa analysis was used to compute inter-observer reliability for subjective IQ scores. Statistical analyses were performed using PASW (version 19.0, SPSS, Chicago, IL, USA) and MedCalc (version 13.3.1.0, MedCalc Software bvba, Mariakierke, Belgium) software.

## Results

### Characteristics of the subjects

In this study, 93 subjects (32, 29, and 32 in groups A, B, and C, respectively) were included. The demographic data for the subjects is summarized in Table 1. The age, height, weight, BMI, body surface area, and average heart rate did not differ significantly between the three groups (*p* > 0.05). Among 93 patients, there were 13 patients with BMI above 25 kg/m^2^, but less than 30 kg/m^2^ (four, three and six patients in groups A, B, and C, respectively). In terms of coronary artery disease, 64 of 93 patients (68.8%) did not show any stenosis, 24 patients (25.8%) had insignificant stenosis (minimal stenosis in 12 patients and mild stenosis in 12 patients), and 5 patients (5.4%) had significant stenosis (moderate stenosis in 4 patients and severe stenosis in 1 patient), respectively.

**Table 1:** Baseline characteristics.

	Group A (n = 32)	Group B (n = 29)	Group C (n = 32)	*p*-value
Age (yrs)	47.88 ± 10.07	51.24 ± 12.54	53.59 ± 11.51	0.136
Sex ratio (female:male)	15:17	11:18	19:13	0.241
Average heart rate (beats per minute)	63.88 ± 12.94	64.17 ± 8.22	65.22 ± 9.82	0.868
Height (cm)	166.10 ± 9.19	162.79 ± 7.35	165.90 ± 8.93	0.251
Weight (kg)	62.97 ± 10.74	59.60 ± 8.51	64.39 ± 10.13	0.161
Body mass index	22.43 ± 2.34	22.45 ± 2.15	23.46 ± 2.23	0.146
Body surface area	1.70 ± 0.18	1.64 ± 0.15	1.72 ± 0.17	0.159
Coronary artery stenosis				
Insignificant stenosis	7 (21.9%)	7 (24.1%)	10 (31.3%)	
Significant stenosis	2 (6.3%)	2 (6.9%)	1 (3.1%)	
Tube voltage (kVp)	100	80	80	
Iodine concentration of contrast media (mg/mL)	400	320	320	
Reconstruction algorithm	FBP	ASIR	ASIR	

Bold *p* value < 0.05. FBP = filtered back projection, ASIR = adaptive statistical iterative reconstruction.

### Radiation dose

There was a significant reduction in the mean radiation dose in groups B and C (2.60 ± 0.48 mSv and 2.72 ± 0.54 mSv, respectively) as compared to group A (3.58 ± 0.67 mSv), which was measured by the estimated effective dose (*p* < 0.001) (Table 2).

**Table 2:** Analyses of objective image quality and radiation dose.

		Group A	Group B	Group C		*p-*value		
					*p*-value	Group A vs. Group B	Group A vs. Group C	Group B vs. Group C
**Dose length product (mGy∙cm)**		255.94 ± 47.69	185.86 ± 33.99	194.26 ± 38.75	**< 0.001**	**< 0.001**	**< 0.001**	0.999
**Effective dose (mSv)**		3.58 ± 0.67	2.60 ± 0.48	2.72 ± 0.54	**< 0.001**	**< 0.001**	**< 0.001**	0.999
**Image Noise**		32.01 ± 10.59	34.01 ± 8.68	34.86 ± 12.62	0.559	0.999	0.882	0.999
**Intravascular Attenuation**	**LM**	469.53 ± 86.63	487.57 ± 88.44	485.58 ± 127.97	0.751	0.999	0.999	0.999
	**LAD**	461.51 ± 93.96	476.58 ± 97.50	471.41 ± 126.00	0.855	0.999	0.999	0.999
	**LCX**	458.04 ± 116.85	467.21 ± 92.34	466.87 ± 123.44	0.936	0.999	0.999	0.999
	**RCA**	436.57 ± 99.08	462.85 ± 96.13	457.73 ± 125.55	0.598	0.999	0.999	0.999
**SNR**	**LM**	16.40 ± 6.92	15.17 ± 4.52	15.34 ± 5.79	0.671	0.999	0.999	0.999
	**LAD**	16.20 ± 7.35	15.05 ± 5.39	14.95 ± 6.22	0.694	0.999	0.999	0.999
	**LCX**	16.14 ± 7.64	14.57 ± 4.27	14.09 ± 4.41	0.349	0.883	0.506	0.999
	**RCA**	15.27 ± 7.02	14.53 ± 5.11	14.75 ± 5.87	0.827	0.999	0.999	0.999
**CNR**	**LM**	19.16 ± 8.46	18.54 ± 5.29	18.98 ± 7.37	0.930	0.999	0.999	0.999
	**LAD**	18.96 ± 8.87	18.43 ± 6.22	18.80 ± 8.39	0.965	0.999	0.999	0.999
	**LCX**	18.90 ± 9.10	17.99 ± 5.03	17.72 ± 6.07	0.790	0.999	0.999	0.999
	**RCA**	18.03 ± 8.53	17.91 ± 5.90	18.08 ± 7.31	0.981	0.999	0.999	0.999

Bold *p-*value < 0.05. Values are mean ± standard deviation. SNR: signal-to-noise ratio; CNR: contrast-to-noise ratio; LM; left main coronary artery; LAD: left anterior descending artery; LCX: left circumflex artery; RCA: right coronary artery.

### Quantitative analyses

**Fig 2:**
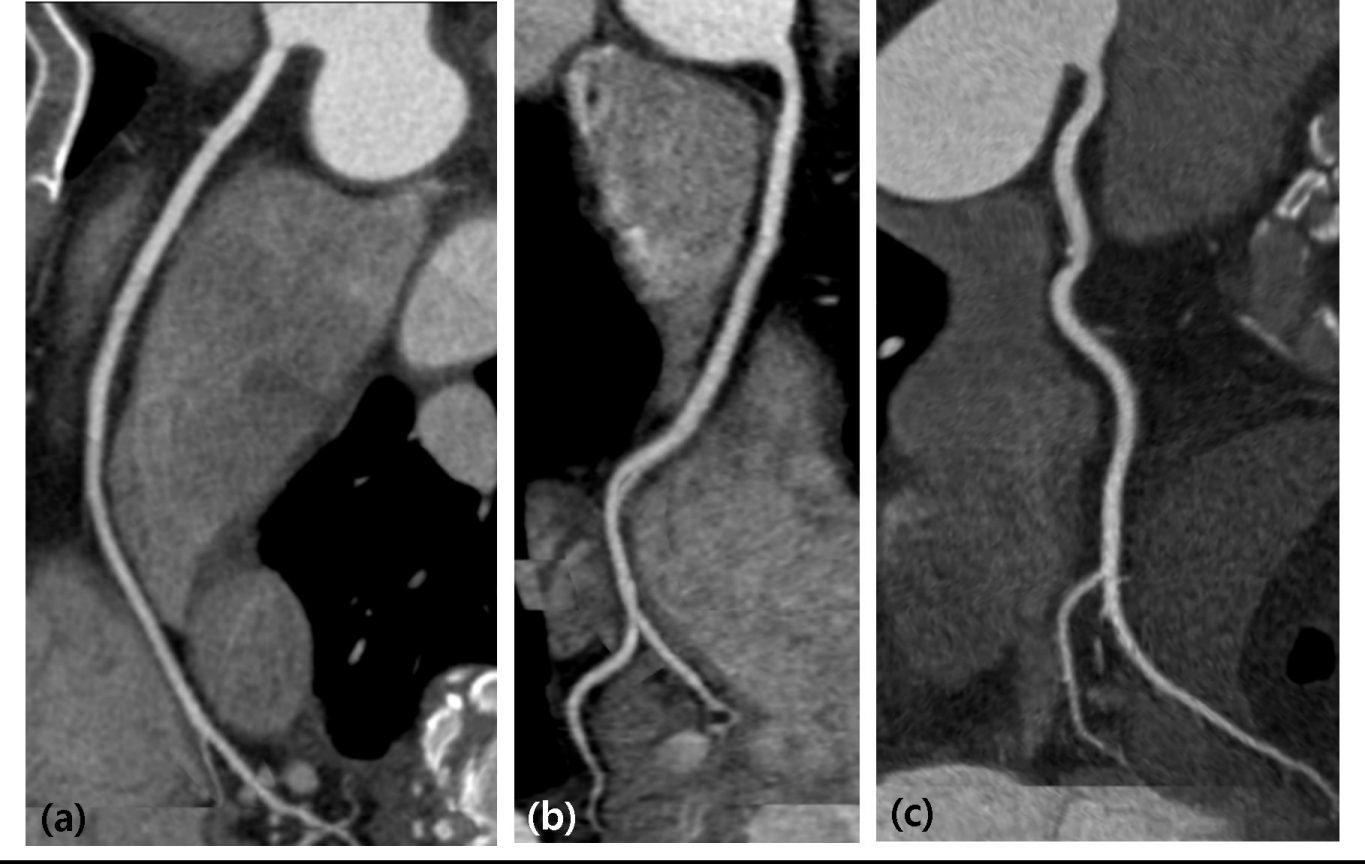
Representative coronary computed tomographic angiography (CCTA) images of groups A, B, and C. Curved multi-planar images of the right coronary artery (RCA) of groups A (a), B (b), and C (c) demonstrating no significant difference in subjective image quality (IQ). (a) CCTA image of a 69-year-old woman (BMI: 20.1 kg/m^2^) with 100 kVp tube voltage, 400 mgl/ml of contrast media (CM) with injection rate of 4 ml/s and reconstructed with filtered back projection. The effective radiation dose was 3.06 mSv and image noise was 26.9 with RCA contrast-to-noise ratio (CNR) of 21.15; (b) CCTA image of a 44-year-old man (BMI: 20.9 kg/m^2^) using 80 kVp tube voltage, 320 mgl/ml of CM with injection rate of 4 ml/s and reconstructed with iterative reconstruction technique. The effective radiation dose was 2.41 mSv and image noise was 27.7 with RCA CNR of 21.30; (c) CCTA image of a 65-year-old man (BMI: 25.3 kg/m^2^) using 80 kVp tube voltage, 320 mgl/ml CM with injection rate of 5 ml/s and reconstructed with iterative reconstruction technique. The effective radiation dose was 2.23 mSv and image noise was 30.3 with RCA CNR of 22.22.

The image noise, intravascular CT attenuation values, SNR, and CNR of the LM, proximal LAD, proximal LCx, and proximal RCA of each group are summarized in Table 2. The image noise and intravascular CT attenuation values tended to be slightly higher in groups B and C than group A, while SNR and CNR tended to be slightly lower in groups B and C than group A. However, the differences were not statistically significant in the three groups (Fig 2). Excellent inter-observer agreement was found for the intracoronary CT attenuation measurements (ICC = 0.900).

We performed subgroup analysis for 13 patients with 25 kg/m^2^ < BMI < 30 kg/m^2^, and found that there was no significant difference in the quantitative measures of coronary arteries according to the tube voltage. However, the SNR and CNR of 80 kVp (Group B and C; n = 9) images with 320 mgl/ml CM and IR tended to be higher than those of 100 kVp (Group A; n = 4) images with 400 mgl/ml CM and FBP, as follows: SNR of LM, 14.36 ± 2.84 vs. 12.89 ± 4.39 (*p* = 0.643), SNR of proximal LAD, 13.76 ± 2.64 vs. 12.53 ± 4.89 (*p* = 0.999), SNR of proximal LCx,13.76 ± 3.17 vs. 11.45 ± 4.36 (*p* = 0.355), SNR of proximal RCA,13.93 ± 3.04 vs. 10.38 ± 3.70 (*p* = 0.090), CNR of LM,17.33 ± 3.93 vs. 14.84 ± 4.63 (*p* = 0.217), CNR of proximal LAD,16.73 ± 3.72 vs. 14.48 ± 4.63 (*p* = 0.355), CNR of proximal LCx,16.73 ± 4.27 vs. 13.40 ± 4.19 (*p* = 0.189), and CNR of proximal RCA,16.90 ± 4.12 vs. 12.32 ± 3.79 (*p* = 0.090), respectively.

### Qualitative analyses

The subjective IQ analyses demonstrated that the scores of vascular attenuation and image noise did not differ significantly between the groups (Fig 2 and Table 3). The non-inferiority of the subjective IQ of groups B and C was revealed, because the upper limit of the two-sided 95% confidence interval (CI) of the mean image noise difference was lesser than the pre-defined non-inferiority margin of 0.6. The values were as follows: for vascular attenuation, group A vs. B, mean difference: -0.117, 0.95% CI: -0.462–0.227, and group A vs. C, mean difference: 0.188, 0.95% CI: -0.185–0.560; for image noise, group A vs. B, mean difference: 0.166, 0.95% CI: -0.266–0.598 and Group A vs. C, mean difference: 0.031, 0.95% CI: -0.375–0.437. The overall inter-observer reliability for subjective IQ analysis was favorable (k = 0.730).

**Table 3:** Subjective image quality analyses.

	Group A	Group B	Group C	*p*-value	*p*-value		
					Group A vs. Group B	Group A vs. Group C	Group B vs. Group C
**Vascular Attenuation**	4.47 ± 0.72	4.59 ± 0.63	4.28 ± 0.77	0.243	0.999	0.883	0.293
**Image Noise**	4.06 ± 0.91	3.90 ± 0.77	4.03 ± 0.70	0.695	0.999	0.999	0.999

## Discussion

The objective of this study was to assess the feasibility of a low-tube-voltage CCTA protocol with low-iodine-concentration CM and IR algorithm. The CCTA at 80 kVp with 320 mgl/ml CM, reconstructed with IR, allowed significant radiation dose reduction without deterioration of objective IQ analyses such as vascular enhancement, SNR, and CNR and showed non-inferiority on subjective IQ, when compared to the protocol at 100 kVp with 400 mgl/ml CM, reconstructed with FBP.

There have been a number of studies comparing image qualities of CCTA using high- and low-iodine-concentration CMs. [11, 13, 14]. However, there is only a few studies which compared the effect of 400 mgl/ml and 320 mgl/ml CMs on CCTA. [11, 15] A study by Becker et al. demonstrated that use of 400 mgl/ml CM is beneficial for coronary arterial enhancement compared with 320 mgl/ml CM at identical scan parameters and contrast flow rates. [11] However, our study showed clinical feasibility of 320 mgl/ml CM without significant difference in the degree of coronary arterial enhancement compared with 400 mgl/ml CM (group A vs. group B), even after the adjustment of iodine delivery rate (group A vs. group C), when the tube potential for CCTA was reduced.

Several studies have shown that iodine delivery rate (iodine flux) is a main determinant of arterial enhancement [10, 16]. In contrast to our expectation that higher vascular enhancement and better CNR might be observed in group C with higher iodine delivery rates as compared to group B, no significant difference was noted in the IQ parameters. We assumed that the main reason could be a higher BMI of the group C subjects (23.46 ± 2.23) than the group B subjects (22.45 ± 2.15) (*p* = 0.044). Notably, our study showed the potential of low tube voltage by increasing intracoronary CT attenuation. Indeed, x-rays generated at 80 kVp were closer to the k-edge of iodine (33.2 keV), maximizing the photoelectric effect with increased contrast enhancement [10, 17, 18].

However, reducing the tube voltage to 80 kVp unavoidably increases image noise, leading to deterioration of IQ. Thus, we adopted the IR technique for noise reduction. The IR algorithm reduces image noise by iteratively comparing the acquired image to a modeled projection [18, 19]. The noise reduction properties of IR permitted the use of low tube voltage with stable image noise and IQ as compared to FBP using high tube voltage, despite the reduction in absolute photon number. Indeed, no significant difference in image noise, SNR, and CNR was noted between high tube voltage using FBP (group A) and low tube voltage using ASIR (group B and C) in our study. Additionally, the low tube voltage CCTA protocol with low-iodine-concentration CM showed non-inferiority in subjective IQ. These results are in concordance with previous studies showing the feasibility of 80 kVp CCTA with iterative reconstruction [20–22]. We suggest that both the amount of iodine administered and radiation dose could be reduced with appropriate IQ, thereby reducing the adverse effects of CM and radiation exposure.

One major drawback of our study was that we did not evaluate the diagnostic accuracy for detecting coronary stenosis by correlating our data with conventional coronary angiograms. As the prevalence of significant coronary artery stenosis was too low (5.4%; five of total 93 patients), we lacked reference standard for the majority of lesions. A recent study by Lee et al. had reported a promising result that CCTA with low-tube-voltage (80 or 100 kVp) and IR enables the accurate non-invasive diagnosis of coronary artery disease, with a sensitivity of 86.4% and specificity of 96.1% in a per segment-based analysis, respectively [23]. There is a need for further investigation regarding diagnostic performance of 80 kVp CCTA with reduced amount of iodine administered., Another limitation of this study was that our study population was confined to the patients with BMI < 30 with relatively low heart rate (< 75 beats/minute). Future studies are necessary to determine the diagnostic performance of CCTA (at 80 kVp and 320 mgl/g of CM) that includes patients of diverse backgrounds with different characteristics. Third, we used a single parameter setting in reconstruction using fixed 50% ASIR blending factors. Fourth, we did not explore the effect of each factor, including tube voltage, iodine concentration, and reconstruction technique, on image quality of CCTA respectively. Instead, we investigate the combined overall effect of low-tube-voltage, low-iodine-concentration, and iterative reconstruction on image quality of CCTA. Lastly, three different types of CCTA acquisition protocols were not compared in the same patients. The subjects were sequentially included and randomly allocated to one of the three arms. Moreover, there were no differences in demographics between the three groups.

## Conclusion

CCTA at 80 kVp with 320 mgl/ml of CM with IR is feasible, achieving reduction of radiation exposure with non-inferior and preserved IQ than at 100 kVp with 400 mgl/ml of CM with conventional FBP.

## Supporting information

S1 File Data of baseline characteristics of the patients and quantitative and qualitative image qualities of CCTA. (XLSX)
